# Understanding the Therapeutic Action of Antipsychotics: Not yet Beyond Striatal Dopamine? A Comment on Direktor et al. (2024)

**DOI:** 10.1093/ijnp/pyae028

**Published:** 2024-07-03

**Authors:** Gavin P Reynolds

**Affiliations:** Biomolecular Sciences Research Centre, Sheffield Hallam University, Sheffield, UK; Rotherham, Doncaster and South Humber NHS Foundation Trust, Doncaster, UK

In their recent Perspectives article, Direktor et al. (2024) rightly draw attention to our poor understanding of both the pathophysiology of schizophrenia and the mechanisms whereby antipsychotic drugs can ameliorate, in part, the behavioral consequences of that pathology. They introduce us to the possibility that the substructure represented by the Islands of Calleja (ICj) in the ventral striatum provides a site of action for antipsychotics through effects, including at dopamine D3 receptors.

This is an intriguing proposition, although some of the evidence they present to support it is open to criticism. Their arguments against the unique role of the dopamine D2 receptors in antipsychotic action include the lack of antipsychotic efficacy of metaclopramide; however, this D2 antagonist is also a high-affinity substrate for p-glucoprotein, preventing its adequate penetration into the brain (Doran et al., 2005). They suggest too that the “atypical” second-generation antipsychotics “bind much less” to D2 receptors. Whether the authors mean that the newer drugs bind more to other receptor sites or have a lower D2 affinity than first-generation drugs—not consistently correct—is unclear. However, it is indisputable that the one factor common to all current antipsychotic agents—the first- and second-generation drugs and the D2 partial agonists—is their ability to bind to dopamine D2 receptors at clinically effective doses.

Nevertheless, the suggestion that dopamine D3 receptor sites are a potentially valuable target for the treatment of schizophrenia is a valid and long-established one (Sokoloff et al., 1992). Direktor et al. (2024) mention clozapine and cariprazine as D3 receptor ligands; several other antipsychotic agents, including amisulpride and blonanserin, also have effects at the D3 receptor implicated in their mechanisms of action and efficacy, although only cariprazine shows a substantially greater affinity for D3 over the D2 receptor. Much has been made of cariprazine’s effect on negative symptoms following the report of its significant advantage over risperidone in patients selected with predominant negative symptoms (Németh et al., 2017). However, in a head-to-head analysis, Leucht et al. (2022) report that risperidone is better than cariprazine in treating the general population of people with schizophrenia; their network meta-analysis (Huhn et al., 2019) also showed risperidone was significantly more effective than cariprazine in reducing positive and overall symptoms in acute treatment. Nor can we extrapolate to a general effect on negative symptoms; cariprazine has no particular advantage over other antipsychotics in its limited ability to ameliorate negative symptoms (Huhn et al., 2019). So, while we cannot rule out a selective benefit of this drug for a small subgroup of people with schizophrenia, it is at best premature and arguably misleading to describe cariprazine as having “therapeutic superiority” (Direktor et al., 2024). Thus there appears to be no consistent evidence for a D3-mediated action in the ICj or elsewhere offering a mechanism to address the negative and cognitive symptoms in most people with schizophrenia.

The authors point out that, in addition to D3 receptors, the ICj have a high density of muscarinic M4 receptors. This is a site of action of xanomeline, an M4 partial agonist, which, formulated with a peripherally acting M1 antagonist, has proven successful in phase 3 trials for the treatment of schizophrenia. Contrary to their description, xanomeline is not the “first nondopaminergic antipsychotic under development”; many compounds previously and currently in development as antipsychotic agents act at transmitter systems without direct effects on dopaminergic function. Furthermore, although not acting at dopamine receptors, xanomeline’s action at striatal muscarinic sites directly influences dopaminergic function, which is why those involved in the early development of the drug described it as dopaminergic (McKinzie and Bymaster, 2012). There are other instances of developmental antipsychotics that are known to modulate striatal dopamine systems being described as “non-dopaminergic”; this description may be an attempt to imply mechanisms reaching beyond the limitations of an effect on dopaminergic function, which is essentially restricted to therapeutic relief of positive symptoms (Reynolds, 2024). While positive symptoms may generally reflect a secondary dopaminergic hyperactivity in the ventral striatum, it seems likely that other neurotransmitter systems are involved in producing the psychotic symptoms in people with schizophrenia not responding to dopaminergic treatments, reflecting the pathological heterogeneity of the disease. Furthermore, it is perhaps time to acknowledge that the negative and cognitive symptoms of schizophrenia need not invoke dopaminergic dysfunction but are likely to be more directly related to the primary pathology, or pathologies, of schizophrenia residing in, for example, hippocampal and neocortical regions.

## Data Availability

There are no data associated with this letter

## Interest Statement

I received no financial support for the research, authorship, or publication of this letter.

I have received research support from Merck and honoraria from Boehringer-Ingelheim and Recordati.
